# Supplementary material for: The stochastic logistic model with correlated carrying capacities reproduces beta-diversity metrics of microbial communities
Source: PLoS Comput Biol. 2022 Apr 1;18(4):e1010043. doi: 10.1371/journal.pcbi.1010043 (PMC9007381; doi:10.1371/journal.pcbi.1010043)
Supplement: S1 Text — OTU selection and fitting a truncated log-normal distribution and estimating the total number of species. Tables with coefficient of determination R2 and correlations between estimated values of K. (PDF) [file pcbi.1010043.s001.pdf]

Supplementary Text

The stochastic logistic model with correlated carrying capacities reproduces beta-diversity metrics of microbial communities

Section A OTU selection

For the determination of the distribution of σ, we include all OTUs for which a positive σ can be estimated from the time-series, and with an occupancy larger than 0.2. We apply this occupancy threshold to avoid an observation bias. In fact, among the very rare OTUs we only observe those with high σ (the others are either not observed or have too low counts to estimate σ and K). Therefore, including OTUs that are too rare would yield a distribution of σ biased towards higher values. Based on previous analyses (see the Supplementary Information of Zaoli & Grilli 2020), we established the threshold at an occupancy of 0.2.

For the determination of the distribution of K, instead, we include all OTUs for which a positive σ can be estimated from the time-series. In this case, in fact, the observation bias is accounted for by fitting a truncated log-normal distribution.

For all the computations of beta-diversity measures, all OTUs are included.

Section B Fitting a truncated log-normal distribution and estimating the total number of species

If the values of K are distributed according to a probability density function $p(K)$ across OTUs, the empirical distribution of K that we compute from the sampled abundances is not exactly $p(K)$. In fact, OTUs with a small K might not be observed, due to the finite sampling depth. There is not a precise threshold under which OTUs are not observed. First, because the average abundance is determined not only by K but also by σ, therefore two OTUs with the same K could have quite different average abundances. Second, because sampling is random. However, I can establish a threshold c such that OTUs with $K > c$ are almost surely observed. This threshold depends both on the sampling depth N_{reads} and on the number of samples. Above this threshold, the measured K will be distributed as the truncated version of $p(K)$:

$$p_{emp}(K) = \frac{\theta(K-c)p(K)}{\int dz \theta(z-c)p(z)}, \tag{1}$$

where $\theta(x-c)$ is the Heaviside function.

For our data, we observe that the measured K are well-described by a log-normal distribution. Their empirical distribution above the threshold c is, therefore,

$$p_{emp}(K) = \sqrt{\frac{2}{\pi s^2}} \frac{1}{K} \theta(K-c) \frac{\exp(-\frac{(\log(K)-\mu)^2}{2s^2})}{\mathrm{erfc}(\frac{log(c)-\mu}{\sqrt{2}s})}, \tag{2}$$

where μ and s are the mean and variance of the normal distribution underlying the log-normal $p(K)$, that is, the mean and variance of the $\log(K)$ (of all OTUs, not just the observed ones). Instead, we call m_1 and m_2 the first two moments of the observed $\log(K)$ above the threshold:

$$m_1 = \frac{1}{S_{obs}} \sum_{i=1}^{S_{obs}} \log(K_i) \tag{3}$$

$$m_2 = \frac{1}{S_{obs}} \sum_{i=1}^{S_{obs}} \log(K_i)^2, \tag{4}$$

where S_{obs} is the number of observed OTUs with $K > c$. The maximum likelihood estimate of the parameters μ and s is given by the following equations:

$$m_1 = \mu + \frac{\sqrt{\frac{2}{\pi}} s \exp(-\frac{(\log(K)-\mu)^2}{2s^2})}{\mathrm{erfc}(\frac{log(c)-\mu}{\sqrt{2}s})} \tag{5}$$

$$m_2 = s^2 + m_1\mu + \log(c)(m_1 - \mu). \tag{6}$$

After having estimated the parameters μ and s , we can estimate the total number of OTUs S starting from the number of OTUs observed above the threshold, using

$$S_{obs} = S * \int_c^\infty dK p(K) = \frac{S}{2} \mathrm{erfc}\left(\frac{\log(c) - \mu}{\sqrt{2}s}\right), \tag{7}$$

which yields

$$S = \frac{2S_{obs}}{\mathrm{erfc}\left(\frac{\log(c)-\mu}{\sqrt{2}s}\right)}. \tag{8}$$

We estimate the parameters μ and s by pooling together the values of K above threshold from all the individuals. The value of the threshold c is chosen such that none of the curves $p_{emp}(K)$ for the different individuals deviate from the fitted log-normal above c. Then, S is estimated for each individual and averaged over individuals. We obtain the following estimates $\mu = -19.85$, $s = 4.93$, and $S = 27616$.

Table A: Coefficient of determination R^2 for Gut communities beta-diversity metrics as predicted by our model.

metric	R^2
Jaccard	0.876
Sørensen	0.865
Whittaker	0.865
Bray-Curtis	0.335
Horn	0.586
Morisita-Horn	0.458

Table B: Spearman correlation cofficients of the values of K estimated over the full-timeseries across individuals of the BIO-ML dataset. All the correlations are highly significant.

	ae	am	an	ao	ba	bc	bh	bj	cl	co
ae	1.00	0.64	0.59	0.57	0.59	0.57	0.58	0.57	0.58	0.58
am	0.64	1.00	0.60	0.55	0.60	0.58	0.59	0.58	0.61	0.58
an	0.59	0.60	1.00	0.55	0.61	0.56	0.59	0.58	0.60	0.60
ao	0.57	0.55	0.55	1.00	0.52	0.56	0.47	0.46	0.51	0.50
ba	0.59	0.60	0.61	0.52	1.00	0.59	0.60	0.62	0.61	0.60
bc	0.57	0.58	0.56	0.56	0.59	1.00	0.55	0.58	0.55	0.55
bh	0.58	0.59	0.59	0.47	0.60	0.55	1.00	0.63	0.62	0.66
bj	0.57	0.58	0.58	0.46	0.62	0.58	0.63	1.00	0.63	0.66
cl	0.58	0.61	0.60	0.51	0.61	0.55	0.62	0.63	1.00	0.66
co	0.58	0.58	0.60	0.50	0.60	0.55	0.66	0.66	0.66	1.00

Table C: Spearman correlation cofficients of the values of K estimated over the full-timeseries across individuals of the Moving Pictures dataset. All the correlations are highly significant.

	M	F
M	1.00	0.62
F	0.62	1.00

Table D: Spearman correlation cofficients of the values of K estimated over the full-timeseries across individuals of the David et al. dataset. All the correlations are highly significant.

	Apre	Bpre	Apost	Bpost
Apre	1.00	0.80	0.54	0.54
Bpre	0.80	1.00	0.55	0.55
Apost	0.54	0.55	1.00	0.57
Bpost	0.54	0.55	0.57	1.00

Table E: Spearman correlation cofficients between the values of K estimated over the first half of each time-series vs the second half. All the correlations are highly significant.

host	correlation
ae	0.65
am	0.75
an	0.76
ao	0.77
ba	0.76
bc	0.77
bh	0.76
bj	0.73
cl	0.8
co	0.78
M	0.76
F	0.76
Apre	0.82
Bpre	0.81
Apost	0.74
