# Supplementary figures and images for: The stochastic logistic model with correlated carrying capacities reproduces beta-diversity metrics of microbial communities
Source: PLoS Comput Biol. 2022 Apr 1;18(4):e1010043. doi: 10.1371/journal.pcbi.1010043 (PMC9007381; doi:10.1371/journal.pcbi.1010043)

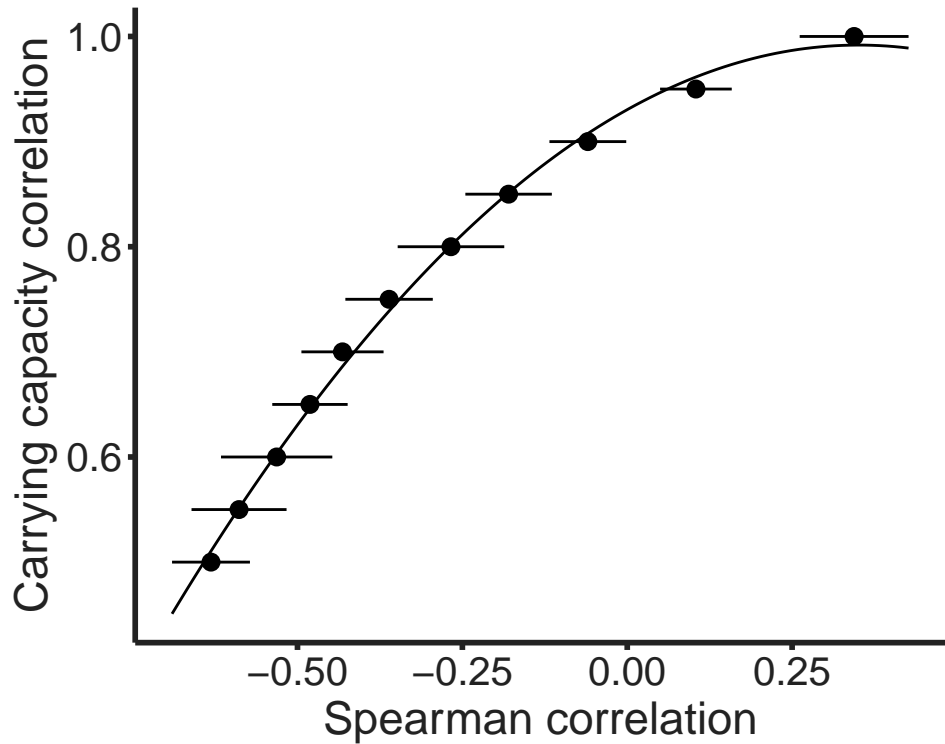

Supplement: S1 Fig — Black points are averages of simulated data (point range corresponds to 2 standard deviations). The black curve is obtained by a quadratic fit to the data, resulting in ρK ∼ 0.92 + 0.34s − 0.48s2, where s is the Spearman correlation. (PDF) [file pcbi.1010043.s002.pdf]
