# Supplementary material for: The stochastic logistic model with correlated carrying capacities reproduces beta-diversity metrics of microbial communities
Source: PLoS Comput Biol. 2022 Apr 1;18(4):e1010043. doi: 10.1371/journal.pcbi.1010043 (PMC9007381; doi:10.1371/journal.pcbi.1010043)
Supplement: Attachment — Submitted filename: ResponseToReviewers.pdf [file pcbi.1010043.s003.pdf]

Response to Reviewers' Comments

Silvia Zaoli Jacopo Grilli

March 4, 2022

Editor's comments

Both reviewers highlight the potential interest in the results, but also raise a number of questions about technical aspects and the presentation of this work. Most of these points seem valid and constructive in making the manuscript more accessible to experts in the field and a broader audience. If you decide to submit a revised version, it is important that you address all of the reviewers' concerns. As suggested by reviewer 1, it would be helpful to shorten the introduction and make the focus of this work clearer.

Reply: We thank the Editor for the positive assessment of our work. We are extremely happy about the quality of the reviews. We edited the manuscript as suggested by the reviewers: we reduced the length of introduction and we included the additional analysis as requested. We also made an effort to remove all the non-essential parts and analysis which went beyond the focus of the work.

Reviewer 1

In their manuscript "The stochastic logistic model with correlated carrying capacities reproduces beta-diversity metrics of microbial communties" the authors state that with a rather simple null-model the change of beta diversity as a function of simple parameters of microbial communities can be predicted. The addressed question is of great relevance in (microbial) ecology. Despite the common usage of beta diversity metrics I agree with the authors that we should better understand where these values actually come from and what impacts them. The text is overall well written and the results are clearly presented. It is always great to find simple rules in complex systems because it may point towards finding something of general importance. However, sometimes you just don't have a real scientific finding but just a general mathematical property. It remains for me a little unclear which of both is the case in the given manuscript..

Reply: We thank the reviewer for the positive remarks and for the suggestions that helped improving the overall quality of the manuscript. We take in deep consideration the last point raised by the reviewer concerning the relevance of simple rules in complex systems. This point falls into the long (interesting, and in some cases fruitful) debate about the roles of patterns and emergence in Ecology (and Life Sciences in general). We feel that this manuscript is not the space to discuss about that (interesting, yet endless) debate. On the other hand, we can take a very pragmatic perspective on our work. If we had to write a program able to generate 'realistic' microbial communities in-silico, what should be the rule of such algorithm? Answering this question has obvious practical (and theoretical) importance. While we do not have a definite answer to the question, we believe that our work add a step in providing a more and more refined answer.

Q 1.1 *Despite being familiar with theoretical modeling I am not a full theoretician myself and several of my raised points might be caused by limited understanding on my site.*

Reply: We made an effort to simplify the description of the theoretical models to make more accessible to a broad audience.

Q 1.2 *1) My major issue with this work is that it remains a little unclear what are "real" findings at all and what are just general mathematical properties without a clear physical statement. The authors state that upon increasing the correlation between the randomly chose Ks the similarity in the obtained communities increases. It seems true for me for every function that as one plugs in more similar values also the outcomes is more similar. So I wonder what the specific modeling approach really tells me beyond this? Is the major statement that it fits the data quantitatively well? But then I feel there should be also more discussion how well the data quantitatively fits the measurements.*

Reply: We thank the reviewer for raising this point. As the reviewer suggests, it is expected that increasing the correlation between the carrying capacities K, the similarity should increase (or, at least, not decrease). This is however not what happens necessarely, as for instance dissimilarity has a non-monotonic relationship with the overlap, as shown in Figure 4. As suggested by the reviewer, we added a Table in the SI which quantitatively measures the agreement between model and data.
Concerning the role of null-models, it is important to remark that the ability in reproducing the overlap-dissimilarity relationship is quite unexpected, given the fact that they were formulated exactly in order to be independent in a ad-hoc null-model. This is another practical consequence of having (null-)quantitative models (i.e., being able to generate communities in-silico): it allows to clarify which observation are actually effect of neglected biological mechanisms (e.g., interactions, in the case of Overlap-Dissimilarity analysis).

Q 1.3 *2) It remains a little unclear how this approach deals with potential regime shifts or more general deviations from steady states. In the introduction it is stated that the model can deal with them but it is not clear to me how. Moreover, regime shifts are removed from the data (infections). So in how far does the model assume that the system stays around a steady state? It also remains a little unclear if the K and σ are obtained for the whole time series or just for parts of it (Fig 1C). How was the time over which K and σ where calculated chosen? Shouldn't the overall length of the timeseries have an impact on these values?*

Reply: We thank the reviewer for this point, which shed light on an aspect that was not well described in the previous version of the manuscript and it was, directly or indirectly, the origin of several of the

points raised by both reviewers. In a previous paper of us (Zaoli and Grilli, Sci. Adv. 2021) we showed that the long term dynamics and the inter-individual differences can be recapitulated in differences (either longitudinal or cross-sectional) of K, while σ has a negligible time/individual variation. In particular, over the timescales of months, the value of K changes abruptly, for multiple OTUs at a time, which we interpret as the macroecological manifestation of regime shifts. Note that these shifts did not correspond to infections (or other external perturbation labeled in the metadata). We clarified our assumptions and their justification based on our previous work in the introduction and the first part of the results/modeling section. Following reviewer #2 suggestions, we also modified that way we compare data and model. In particular, we now infer ρ_K from the data and compare it with the prediction of ρ_K obtained with the model.

Q 1.4 *3) As stated by the authors sequencing data does not result in absolute abundances and therefore does not give the carrying capacities. Within a sample the measured steady state populations densities (reads) may be in the same way proportional to K but across samples this is not true. Accordingly I wonder how K from different samples can be compared (Fig1A)?*

Reply: We agree with the reviewer on this point. The proportionality constant between carrying capacity and K could vary across samples. This fact does not however impact the estimates of the distribution of K (see also Grilli 2020 Nat. Comm. in the Supplementary Materials sec S2B regarding this point).

Q 1.5 *4) The authors assume that two communities from the same environment have the same σ, but different values of K. I don't understand this. Aren't K and σ estimated for all the OTUs within a community? What does K and σ of a community then mean? Average across all OTUs? Why is K different and σ constant across communities?*

Reply: The text was unclear and we edited it. Each OTU i is characterized by a value σ_i. The question we were discussing is whether this value change, for that OTU, across community or not. This choice is motivated by the results of Zaoli & Grilli, Sci Adv 2021.

Q 1.6 *5) For the sampling two correlated values of K are drawn from a Gaussian distribution with a certain correlation. It remains for me a little unclear in how far this correlation argument is justified. Data is just shown for the correlation between two individuals in Fig 1D. That seems quite little data to make an assumption that is so central for the paper.*

Reply: We added tables of the correlations across (and within) individuals in the SI. This choice is again motivated by the results of Zaoli & Grilli, Sci Adv 2021, where it is shown that both the variability across OTUs of K and the variability of each K_i over time and across individuals are Lognormally distributed and combine multiplicatively. This fact implies that each pair of $\log K_i$ in two communities is, in full generality, distributed as a bivariate gaussian.

Q 1.7 *6) The authors state that their model explains the overlap-dissimilarity connection that can often be found but doesn't have to be there theoretically. However, isn't that simply a result of a detection threshold? If there is a detection threshold than for highly correlated K the two sampled species are either both above or below the threshold and thus either appear together or not with similar population densities e.g. high overlap and low dissimilarity. If K is less correlated the dissimilarity will increase because population densities become more different and it is also more likely that just one of the two falls below the detection threshold. Is there a way to better understand where this overlap-dissimilarity connection comes from?*

Reply: Yes, the reviewer is correct in writing that the negative relationship (and the positive one at low values of overlap) are consequence of finite sampling. To be more precise, in our model, for infinite sampling efforts, the values of the overlap converge to 1. The finitness of sampling is however integral part of both data and model. We further clarify this in the text.

Q 1.8 *.. are present at a given locations → locations*
I think it would be very useful to give line number for a manuscript otherwise it is rather difficult to refer to specific sentences.

Reply: Edited as suggested.

Q 1.9 *What exactly is plotted in Figure 1? Are these binned histograms? Why is $P(K)$ reaching around 1 at the maximum? Shouldn't it be much smaller than that given that the integral over the plot should equal 1?*

Reply: Yes, the are binned histograms. They are normalized so to match probability distributions. If $h(x_i)$ is the number of observations in the bin of values between x_i and $x_i + dx$, we plotted $h(x)/Z$ where $Z = \sum_i h(x_i)dx$. We clarify this in the text.

Q 1.10 *In Fig2: 100 values have the same K for both samples. Shouldn't that result in a massive black blob at correlation=1 ?*

Reply: No, because even if all the OTUs in two samples have exactly the same values of K ($\rho_K = 1$), their abundance are also determined by the (independent) fluctuations of the Stochastic Logistic Model. We clarified this in the text.

Q 1.11 *I feel the text – especially the introduction – could be more concise and to the point. A lot of ideas seem to be introduced that seems not so central to the actual work. One has to read quite some text to find out what the goal of the paper actually. It's of course the authors choice what they write, but I feel a little more focus would help.*

Reply: Thanks. We focused the introduction as suggested.

Q 1.12 *In the supplementary figures are several times questions marks where probably should be numbers.*

Reply: Edited as suggested.

Reviewer 2

In the manuscript entitled "The stochastic logistic model with correlated carrying capacities reproduces beta diversity metrics of microbial communities", Zaoli and Grilli build the null model for microbial communities using the stochastic logistic model and analyze some metrics of beta diversity. Their null model can show some patterns of beta diversity in empirical studies such as Overlap-Dissimilarity relations.
I think Result D and Figure 4 (i.e., the relationship between Overlap and Dissimilarity) are very interesting. However, I think the rests of the results need to be clarified and revised..

Reply: We thank the reviewer very much for the positive remarks.

Q 2.1 *1 In the current draft, it is unclear why the authors call their model the null model. I guess because the model does not include any species interactions and any other ecological properties such as migration, but please explain clearly what the authors mean by null in Introduction.*

Reply: Thanks for pointing this out. We decided to remove 'null', as, in some sense, every model does not include all the details and is therefore 'null' respect to the neglected mechanisms. We feel that in this context, the wording 'null' might be confusing.

Q 2.2 *2 Result A and Figure 1 2.1 The current figures 1 a and b do not explain what the color dots and lines represent and how they are calculated. This point prevents my understanding in section Result A.*

Reply: Both points and lines are (normalized) histograms of the empirical values of K and σ in the data. We clarified this in the text.

Q 2.3 *2.2 In the main text, the authors argue that "as shown in Fig.1 C, the estimation of K . . . are strongly correlated" (the first paragraph of Result A) and that "however the correlation is lower than the temporal one (Fig 1D)" (the second paragraph of Result B), they do not show any correlation coefficients. I think that they should show for example Pearson or Spearman correlations.*

Reply: We added tables in the SI reporting the correlation coefficients between K when they are estimated along time-series. We included coefficients across individuals and within two parts of each time-series.

Q 2.4 *3 Result B: I have some concerns in this section.*
3.1 First, the section title seems misleading. In this section, the authors show the scatter plots of Pearson correlations of species abundances between two environments and other metrics of beta diversity obtained from their null model. The authors do not predict anything here.

Reply: Thanks for pointing this out. We edited this part, as the rational was unclear. One challenge is that the value of ρ_K between two samples is unknown. We now show (in the Methods and SI) that it is possible to estimate the value of ρ_K using the Spearman correlation of abundances between the two samples. In Figure 3, we estimate ρ_K for the samples and then compare with the prediction of the model for the corresponding value of ρ_K.

Q 2.5 *3.2 Second, the choice of the metrics of beta diversity seems problematic. Although some measures (e.g., Bray-Cruits dissimilarity and Jaccard similarity) are widely used in (microbial) ecology, I have never seen the studies using Pearson correlation as beta diversity (see also the next comment). In addition, there are many metrics of beta diversity that the authors do not analyze but are widely used (e.g., Sørensen index, the ratio of gamma diversity to alpha diversity, or gamma diversity minus alpha diversity). See, for example, Beck et al (2013) https://onlinelibrary.wiley.com/doi/10.1111/2041-210x.12023, Tuomisto (2010), https://onlinelibrary.wiley.com/doi/10.1111/j.1600-0587.2009.06148.x, or Barwell et al (2015), which the authors cite (ref. 24). The authors need to reconsider the choices of the metrics and/or to justify them (e.g., choosing metrics that are widely used in recent studies).*

Reply: Thanks for pointing this out. We modified the choice of beta-diversity as suggested and we now included more commonly used ones.

Q 2.6 *3.3 Related to the above two points, I am not sure whether the x-axes of figure 2 (and figure 3) should be Pearson correlation of species abundances. If the authors want to predict something, I think they should plot the (each measure of) beta diversity in the empirical data against the beta diversity predicted by the null model tuning parameter* ρ_K. *As I do not think Pearson correlation is used as beta diversity, figure 2 (and figure 3) does not make sense to me.*

Reply: We changed the x-axis to the value of ρ_K as suggested in both figure 2 and 3

Q 2.7 *4 In addition to the above comment, I have a concern about figure 3 and the sentence "The relationship predicted by the null model follows in a remarkably precise manner the empirical patterns" in the first paragraph of section Result C. Of course, the pattern of the mean values in the null model (red dots in figure 3) seems similar to the patterns of empirical data. However, if we compare the exact points from the null model (i.e., figure 2) with the empirical ones (figure 3), I am not sure whether the distributions of dots in the null model and empirical data are similar. It seems to me that the distributions of the dots at least in panels B, C, and D differ between figures 2 and 3. The authors need to quantify the similarities between the null model and the empirical data.*

Reply: Thanks for raising this point. We modified the sentence and added a table in the SI that reports the values of $R2$ obtained with our predictions. It is important to notice that in this work we limit ourselves to the average/typical value of beta-diversity. Predicting the full distribution would require an exact knowledge of the value of ρ_K for each pair of samples and, most likely, the inclusion of mechanisms (e.g., interactions and other sources of species-species correlations) which are explicitly neglected in our setting. We added a short paragraph in the discussion regarding this point.

Q 2.8 *Minor comments*
1. Figure legend of 1a. I recommend that the authors add the explanation on the dashed line. Although it is explained in the main text, the figure legend should be (ideally) clear without reading the main text. In addition, the figure legend should explain what the colored dots and lines represent in panels A and B.
2. I think there is a typo in r.h.s. of equation (2): $1/(\tau) * \lambda * (1 - \lambda/K) + \dots$
3. In Method D and Result B, adding the indexes to the beta diversity measurements could make the first sentence clearer. I mean (i)Pearson correlation, (ii) Jaccard similarity index, (iii) Morisita-Horn dissimilarity, (iv)Brays-Cruits dissimilarity .... In addition, Result B says the authors analyze seven metrics while Method D says they analyze six measures. This is inconsistent.

Reply: Thanks. We edited as suggested.
